# Single nucleotide variant in coenzyme Q6 reprograms macrophage metabolic remodeling in response to inflammatory signals

**DOI:** 10.21203/rs.3.rs-9986029/v1

**Published:** 2026-06-22

**Authors:** Xue Lin, Elizabeth M. Todd, Edgar P. Anaya, Sharon Celeste Morley

**Affiliations:** Washington University in St. Louis; Washington University in St. Louis; Washington University in St. Louis; Washington University in St. Louis

**Keywords:** coenzyme Q6, immunometabolism, host defense, Streptococcus pneumoniae, macrophage, inflammation

## Abstract

Macrophage metabolic remodeling sustains inflammatory responses to pathogens. At homeostasis, macrophages rely on oxidative phosphorylation (OXPHOS), but during inflammation, OXPHOS is downregulated and aerobic glycolysis increases. Increased flux through the tricarboxylic acid (TCA) cycle increases the availability of substrates, such as succinate, that promote pro-inflammatory transcription. While metabolic remodeling has been extensively characterized, the mechanisms governing the shift from OXPHOS to glycolysis remain unclear. We recently identified a single nucleotide variant (SNV) in a mitochondrial protein, coenzyme Q6 (COQ6), that accelerates OXPHOS downregulation during infection with the Gram-positive organism *Streptococcus pneumoniae*. Because the SNV converts an aspartate residue (D) to tyrosine (Y), we denote the variant as *COQ6*^*DY*^. Here, we now systematically compare the inflammatory responses of macrophages expressing *Coq6*^*DY*^ or *Coq6*^*WT*^ after stimulation with the *S. pneumoniae* -derived pneumolysin (PLY), or with lipopolysaccaharide (LPS; a toxin derived from Gram-negative bacteria). We found that *Coq6*^*DY*^ reprograms macrophage mitochondrial metabolism by changing the balance between OXPHOS and glycolytic activity and relative TCA metabolite concentrations at homeostasis. Furthermore, responses to PLY varied by host genotype and by concentration of available glucose, whereas responses to LPS were less dependent upon genotype. We therefore identify a non-canonical function of COQ6 in governing mitochondrial metabolism.

## Introduction

Effective immune responses require remodeling of macrophage metabolism ^[1]^. At rest, macrophages generate ATP primarily through oxidative phosphorylation (OXPHOS); upon stimulation with lipopolysaccharide (LPS), macrophages downregulate OXPHOS and upregulate aerobic glycolysis. The rebalancing of OXPHOS and glycolysis is referred to as metabolic remodeling. The current paradigm suggests that upregulation of glycolysis promotes and maintains an inflammatory phenotype through increased production of tricarboxylic acid (TCA) intermediates such as succinate and fumarate ^[2, 3]^. The molecular mechanisms driving metabolic remodeling after receipt of an inflammatory stimulus are not yet fully defined.

We recently described a novel single nucleotide variant (SNV) in coenzyme q6 (encoded by the gene *COQ6*) that alters inflammatory macrophage metabolic remodeling and increases susceptibility to lung infection with S*treptococcus pneumoniae* (pneumococcus) ^[4]^. COQ6 is a monooxygenase localized to the inner mitochondrial membrane and is one of at least eleven enzymes required to synthesize the lipid ubiquinone (also called Coq10). Ubiquinone acts as an electron receptor in the electron transport chain, accepting electrons from complexes I or II. Previously identified pathologic variants in *COQ6* impair ubiquinone biosynthesis and present with various multi-organ syndromes, often including renal disease.

We identified the novel SNV through whole exome sequencing of a pediatric population highly susceptible to pneumococcal pneumonia in Papua New Guinea. The SNV converts an aspartate (D) residue to tyrosine (Y) at position 308 in human *COQ6* and at position 316 in murine *Coq6*, and we thus denote the protein variant as COQ6.DY, or simply DY ^[4]^. A mouse model expressing the homologous variant in murine *Coq6* was generated, and homozygous variant mice (DY mice) demonstrated increased mortality after *S. pneumoniae* challenge, confirming causality. Bone marrow-derived macrophages (BMDMs) derived from DY mice showed impaired intracellular killing of *S. pneumoniae* ^[4]^. In contrast to previously identified pathogenic *COQ6* variants, *Coq6*^DY^ did not reduce ubiquinone production. Instead, alveolar macrophages and cardiomyocytes from DY mice revealed accelerated downregulation of OXPHOS after exposure to either *S. pneumoniae* or the pneumococcal toxin, pneumolysin (PLY) ^[4]^. We thus identified the remodeling of mitochondrial metabolism after exposure to an inflammatory stimulus as a secondary, or non-canonical, function of COQ6.

We undertook the current study to clearly define how *Coq6*^DY^ regulates mitochondrial metabolism by quantifying metabolic switching in BMDMs from WT or DY mice under inflammatory conditions. We selected BMDMs as a well-characterized, commonly used primary macrophage that could be generated in sufficient quantities for analyses. We characterized OXPHOS, glycolysis, and tricarboxylic acid (TCA) metabolites at homeostasis or after exposure to two different inflammatory stimuli, lipopolysaccharide (LPS) or PLY. LPS is commonly used to stimulate inflammation *in vitro*, and exposure to LPS is sufficient to induce metabolic switching. However, LPS is found only in Gram-negative bacteria and does not model inflammation driven by Gram-positive organisms. We therefore also used PLY, a major toxin derived from *S. pneumoniae*, as an inflammatory stimulus. Unlike LPS, PLY is a pore-forming toxin and can lyse cells at high concentrations. At sub-lytic concentrations, PLY induces inflammatory signaling and NLRP3 activation. To capture metabolic switching during inflammation, we measured OXPHOS, glycolysis, and TCA cycle intermediates. To characterize inflammatory cell responses that correlated with metabolic changes, we assessed cytokine production, concentrations of NAD + and NADH, and neutralization of H_2_O_2_ generated by *S. pneumoniae*. Because tissue glucose concentrations drop precipitously during bacterial infections ^[5]^, we compared outcomes in the presence or absence of glucose. Our results demonstrate that macrophage metabolic remodeling depends upon the inflammatory stimulus, glucose availability, and *Coq6* genotype.

## Methods

### Mice.

Mice homozygous for the *Coq6*^*DY*^ variant were generated by CRISPR-Cas9 mutagenesis in embryonic stem cells derived from C57Bl/6 mice and back-crossed to B6 twice to reduce likelihood of off-target effects, and are fully described in a prior report ^[4]^. In all experiments, WT mice are derived from the same CRISPR/back-cross lineages, maintained in our animal facility, and matched for age (within 2 weeks) and biological sex. We used both male and female mice as sources of bone marrow-derived macrophages in our experiments. Mice were used between 8 and 12 weeks of age, and the weight of females and males were 18–22g and 22–25g, respectively. All animals were bred in-house and maintained in specific pathogen-free barrier facilities supervised by the Division of Comparative Medicine (DCM) at WashU. All barrier facilities maintain a diurnal 12:12 (hours) light:dark cycle with temperatures ranging from 20–26°C and humidity ranging from 30–70%. In accordance with current guidelines from the Panel on Euthanasia of the American Veterinary Medical Association, animals were euthanized with carbon dioxide using a “SMARTBOX,” or Rodents CO_2_ Euthanasia System, provided by the Division of Comparative Medicine at WashU. These CO_2_ Euthanasia Systems fill the chamber at a constant rate of 30%−50% volume per minute to minimize distress to the animals. Death was confirmed by physical examination.

### Ethics declaration (animal subjects).

Animal experiments were performed at WashU in accordance with the Animal Study Protocol (# 21–0322) approved by the WashU Institutional Animal Care and Use Committee, Animal Welfare Assurance #D16–0245.

### Generation of bone marrow-derived macrophages.

Bone marrow-derived macrophages (BMDMs) were generated from the bone marrow of WT and DY mice as previously described ^[4]^. In brief, bone marrow was collected via centrifugation of femurs and/or tibiae of WT or DY mice. After erythrocyte lysis (BD PharmLyse; BD Biosciences), remaining cells were incubated in complete DMEM media (DMEM (Thermo Fisher Scientific) 10% FBS, 1% pen/strep, 1% HEPES pH 7.2, 1% GlutaMAX) with 10% L929-conditioned medium. Media was exchanged every 2–4 days and BMDMs used for experiments on day 7.

### Inflammatory stimuli.

BMDMs were stimulated *in vitro* with LPS 500 ng/mL (Sigma Aldrich L4391); PLY 500 ng/mL (LSBio); or *S. pneumoniae* D39 (MOI 20:1) for the indicated duration.

### Analysis of mitochondrial metabolism.

Macrophage mitochondrial metabolism in BMDMs was quantified using commercially available kits (Agilent). The Mito Stress kit measures oxygen consumption (OCR) and extracellular acidification (ECAR) while components of the electron transport chain are inhibited, providing quantification of parameters such as ATP-linked OCR, basal OCR, and maximum OCR. The Glycolysis Stress test measures glycolytic capacity after a brief period of glucose starvation. The Mito Fuels Flex test measures the dependence and capacity of mitochondria to oxidize glutamine, long chain fatty acids, or glucose during OXPHOS. BMDMs were plated in cDMEM at a density of 5 × 10^4^ cells per well in Agilent Seahorse XF96 culture microplates (Agilent Technologies) and rested overnight (37°C). On the day of the assay, medium was replaced with fresh XF DMEM containing substrates required for the specific assay, according to manufacturer’s instructions. The Seahorse Mito Stress Test was performed by measuring OCR and ECAR after sequential injection of inflammatory stimuli (LPS 500 ng/mL or PLY (500 ng/mL), oligomycin (2 μM), FCCP (5 μM), and a rotenone/antimycin A cocktail (0.5 μM) in complete XF DMEM. Seahorse XF Glycolysis Stress Test was performed by measuring ECAR and OCR after sequential injection of inflammatory stimuli (LPS 500 ng/ml or PLY (500 ng/ml)), glucose (5 μM), oligomycin (2 μM), and 2-DG (50 mM). Seahorse XF Mito Fuel Flex Test was performed by measuring OCR of BMDMs in the presence or absence of fuel pathway inhibitors, BPTES (3 μM), Etomoxir (4 μM), and UK5099 (2 μM). Sequentially inhibiting the pathway of interest followed by the two alternative pathways enables the calculation of BMDMs’ mitochondrial dependency to meet energy demand; and inhibiting the two alternative pathways followed by the pathway of interest enables the calculation of BMDMs’ mitochondrial capacity to meet energy demand. In each assay, experimental conditions were measured in technical replicates (≥ 3 wells/condition). Data were acquired using Seahorse Wave Controller Software v 2.6. The assay cycle from which values were derived is denoted by a subscript, *i.e*. “OCR_4_” means the OCR measured during the fourth cycle of the analysis.

### Analysis of TCA intermediates

Relative concentrations of TCA metabolites were measured by mass spectrometry conducted by the WashU Diabetes Research Center core facility. BMDMs were derived from at least three WT or DY mice and pooled for analysis. After stimulation, cells were snap frozen and submitted for mass spectrometry. Prior to mass spectrometry, cells (4 × 10^6^/mL per sample) were suspended in water. TCA metabolites, along with itaconate, glutamate, and glutamine were extracted from 0.05 mL of suspension with protein precipitation. The pyruvate, α-ketoglutarate, fumarate, succinate, oxaloacetate, citrate, malate, isocitrate, and itaconic acid were derivatized with O-benzylhydroxylamine to improve the liquid chromatography separation. Quality control (QC) samples were prepared from pooled study samples and injected every 10 study samples to monitor intra-batch precision. The pyruvate, α-ketoglutarate, fumarate, succinate, oxaloacetate, citrate, malate, isocitrate, itaconic acid, Glu, and Gln were analyzed on a 4000QTRAP mass spectrometer coupled with a Prominence LC-20AD HPLC system. Data processing was conducted with Analyst 1.6.3. Relative quantification data were reported as the peak area ratio of analytes to their respective internal standards. Only metabolites with coefficient of variation < 15% in QC injections were reported.

### Analysis of cytokine production

After stimulation with indicated inflammatory stimuli, supernatants were harvested from cultures of BMDMs and stored at −80°C for measurement later. Cytokine and chemokine concentrations in the supernatant were determined by Aimplex assay, according to manufacturer instructions (Aimplex Biosciences).

### Immunoblot

After exposure to the indicated inflammatory stimulus, BMDMs were washed twice with ice-cold PBS, then incubated on ice with ice-cold RIPA buffer with 1mM PMSF, 1mM Na3VO4, and 1mM NaF (250 μl per 3–3.5 × 106 cells) for 30 min. Cell lysates were centrifuged at ~ 14,000 g × 15 min. Supernatants were transferred to new 1.5 mL tubes, and 2x Laemmli sample buffer was added. The lysates were boiled at 95–100°C for 10 min and stored at −20°C for future analysis. Immunoblots were performed as described ^[6]^. Membranes were probed with the indicated primary antibodies, anti-SDHA (1:500, Cell Signaling Technology, 5839S), anti-IDH3A (1:500, Invitrogen, PA5–142769), anti-Glutamate Dehydrogenase (1:500, Cell Signaling Technology, 12793S), and anti-OGDH (1:500, Invitrogen, PA5–106306). Beta-actin (1:1000, Cell Signaling Technology, 8457S), GAPDH (1:2000, Cell Signaling Technology, 2118S0, and Alpha-tubulin (1:1000, Cell Signaling Technology, 2144S) were used as loading controls. Goat anti-rabbitAlexaFluor680 (Invitrogen) antibodies were used to detect membrane-bound antibodies. Signals were visualized using the LiCOR Odyssey imaging system and software (LiCOR, Lincoln, NE).

### SDH Activity

SDH activities were detected with SDH Activity Assay Kit (Novus Biologicals, NBP3–25801). All measurements and data analysis were performed according to the manufacturer’s instructions.

### NAD+/NADH Quantification

Quantification of NAD + and NADH were measured with NAD+/NADH Assay Kit (Sigma-Aldrich, MAK468), according to the manufacturer’s instructions, 60 min after addition of indicated stimulant.

### H_2_O_2_ Concentrations

*S. pneumoniae* (serotype D39) was added to cultures of BMDMs at m.o.i. of 1:20. After incubation for 60 min at 37°C, H_2_O_2_ concentrations were detected with Thermo Scientific Pierce Quantitative Peroxide Assay Kit (Thermo Scientific, 23280). All measurements and data analysis were performed according to the manufacturer’s instructions.

### Statistics

All data were stored as either Microsoft Excel or GraphPad Prism spreadsheets. Normality of data was tested using the Shapiro Wilk test. Comparison of two groups of normally distributed data was performed using student’s t-test (two-tailed, unpaired). Non-parametric Mann-Whitney (two-tailed) was used to compare two groups of non-parametric data. ANOVA (normally distributed data) or Kruskal-Wallis (non-parametric data) tests with follow-up pair-wise comparisons (Holm-Šídák or Dunn’s, respectively) adjusted for multiple comparisons were used to compare values from > 2 groups. When noted in legends, values were paired by experiment (biological replicate) for analysis to account for inter-experiment variation. P-value of < 0.05 was considered statistically significant. All experiments were independently performed using biological replicates at least twice. The number of technical replicates is included in each figure legend.

## Results

**Figure 1:**
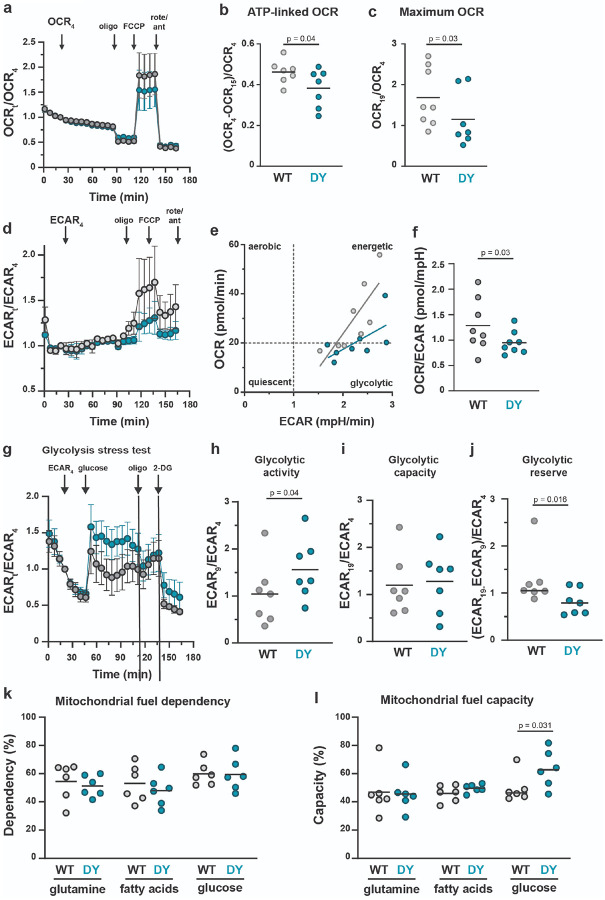
Unstimulated BMDMs from DY mice exhibit reduced OXPHOS and greater glycolytic activity. **(a)** Normalized OCR of unstimulated BMDMs derived from WT (grey) or DY (blue) mice measured using the Seahorse XF Cell Mito Stress Profile. BMDMs were incubated in XF DMEM media with 10mM Glucose, 1mM pyruvate, and 2mM glutamine. **(b)** Normalized ATP-linked OCR, and **(c)** normalized maximum OCR were derived from profiles shown in (a). **(d)** Normalized ECAR, **(e)** Bioenergetic profiles, and **(f)** OCR_4_:ECAR_4_ ratio of unstimulated BMDMs derived from WT (grey) or DY (blue) mice, treated as in (a). Bioenergetic profiles generated by plotting absolute OCR_4_ and ECAR_4_ values obtained in 7 independent assays. Simple linear regression of WT (gray line; slope 2.5 and significantly non-zero (p = 0.02), y-intercept 5.9, r^2^ = 0.7,) or DY (blue line; slope 1.4 and not different from zero, y-intercept 4.0, r^2^ = 0.43) values are shown; the y-intercepts are significantly different (p = 0.01). **(g)** Seahorse XF Cell Glycolysis Stress profiles, **(h)** glycolytic activity, **(i)** glycolytic capacity, and **(j)** glycolytic reserve of unstimulated BMDMs derived from either WT (gray) or DY (blue) mice. ECAR was serially measured over time with substrates/inhibitors added at indicated times. **(k)** Mitochondrial fuel dependency and **(l)** capacity of BMDMs derived from WT (gray) or DY (blue) mice determined by Agilent Seahorse Mito Fuels Flex test. **(a, d, g, k)** OCR or ECAR at each time point (OCR_t_ or ECAR_t_) is normalized to OCR_4_ or ECAR_4_ (obtained during fourth cycle). Arrows indicate time when inhibitors were added. Symbols represent mean ± SEM of 4 (a,d) or 6 (g,k) independent experiments. **(b, c, e, f, h, i, j)** Each symbol represents the average of technical duplicates obtained in 7 or 8 independent assays. Data show values with line at mean, analyzed by paired Student’s t-test., for normally distributed data (b,c,f,h) or line at median, analyzed by Wilcoxon rank test, (j,l) for non-parametric data.

We first defined resting metabolism in unstimulated BMDMs from WT or DY mice by comparing baseline reliance on OXPHOS and glycolysis (Fig. 1). We quantified the oxygen consumption rate (OCR) and extracellular acidification rate (ECAR) using the Agilent Seahorse Mito Stress test. BMDMs were incubated with multiple energy substrates (glucose (10 mM), pyruvate (1 mM) and glutamine (2 mM); Fig. 1a–f). BMDMs from DY mice exhibited reduced maximal OCR and reduced ATP-linked OCR (Fig. 1a, b, c). Simultaneous acquisition of ECAR (representing glycolysis) revealed that BMDMs from DY mice had an overall energy profile that favored glycolysis relative to OXPHOS. Specifically, the ratio of OCR:ECAR, quantified as a proxy for OXPHOS:glycolysis, was significantly lower in BMDMs from DY mice (Fig. 1d, e, f).

To further analyze glycolytic activity, we compared the induction of glycolysis after glucose re-feeding in WT and DY BMDMs using the Seahorse Glycolysis Stress test (Fig. 1g–j). ECAR was measured after BMDMs were briefly (30 min) deprived of pyruvate and glucose, after which a saturating concentration of glucose was injected (Fig. 1g). DY BMDMs again demonstrated greater glycolytic activity than WT (Fig. 1h). Total glycolytic capacity in WT and DY BMDMs was equivalent (Fig. 1i). With equivalent glycolytic capacity but increased glycolytic activity at homeostasis, unstimulated DY BMDMs exhibited reduced glycolytic reserve (Fig. 1j).

We then measured the dependence on and capacity for generating ATP from glucose, glutamine, or fatty acids in WT or DY BMDMs using the Agilent Seahorse Mito Fuels Flex test. Dependency on a fuel source indicates that the cells’ mitochondria cannot compensate for the blockade of one fuel source by oxidizing the other available fuels. Capacity of a fuel source measures the ability of mitochondria to meet cellular energy demand using one fuel when the other two fuels are unavailable. There was no difference between WT and DY BMDMs in dependency upon glucose, glutamine or fatty acids (Fig. 1k), but DY BMDMs showed greater capacity to use glucose (Fig. 1l).

**Figure 2:**
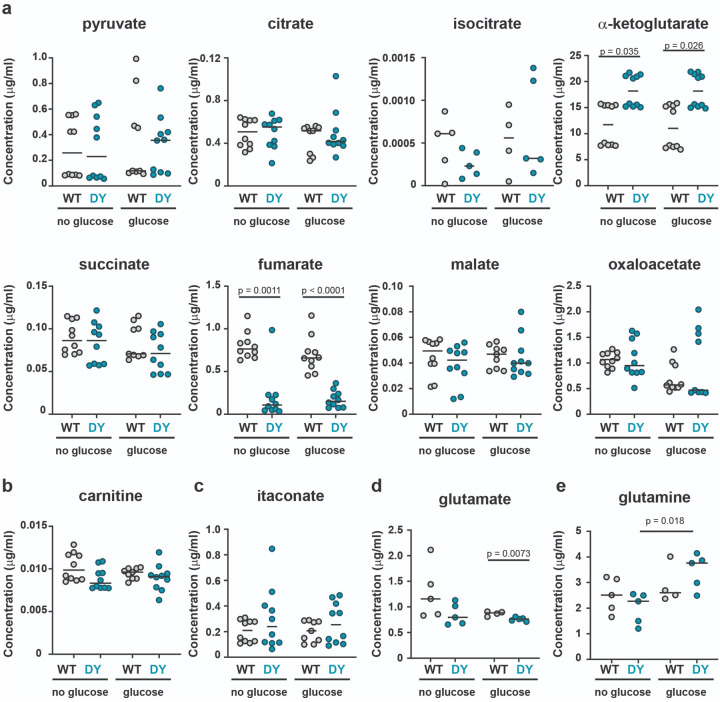
Key TCA metabolites correlate with altered mitochondrial metabolism in BMDMs from DY mice. **(a)**Concentrations of pyruvate and TCA cycle intermediates, (b) carnitine, (c) itaconate, (d) glutamate, or (e) glutamine in unstimulated BMDMs derived from WT (gray) or DY (blue) mice, incubated with or without glucose. Each symbol represents value of one of five replicates of BMDMs pooled from at least three mice, analyzed in two mass spectrometry runs. Line at mean for normally distributed data or median for non-parametric data. Four groups in each graph were analyzed by either ANOVA followed by pairwise comparison (normally distributed) or by Wilcoxon ranked tests (non-parametric data), followed by pairwise comparison of pre-selected groups: WT vs DY no glucose; WT vs DY with glucose; WT no glucose vs WT with glucose; DY no glucose vs DY with glucose. Significant p-values between paired comparisons are shown.

We next tested if the differences in OCR, glycolytic activity and glycolytic capacity found in DY BMDMs were associated with altered concentrations in pyruvate, TCA metabolites (Fig. 2a), carnitine (Fig. 2b), itaconate (Fig. 2c), glutamate (Fig. 2d) or glutamine (Fig. 2e). We measured metabolite concentrations in the presence or absence of glucose. Regardless of glucose availability, concentrations of α-ketoglutarate were significantly higher in BMDMs from DY mice, while concentrations of fumarate were significantly lower (Fig. 2a). When glucose was available, BMDMs from DY mice exhibited lower concentrations of glutamate compared to WT (Fig. 2d). Glucose availability significantly increased the concentration of glutamine in DY BMDMs, but not in WT (Fig. 2e). Other metabolites assayed did not vary between WT and DY cells or change with glucose availability.

**Figure 3:**
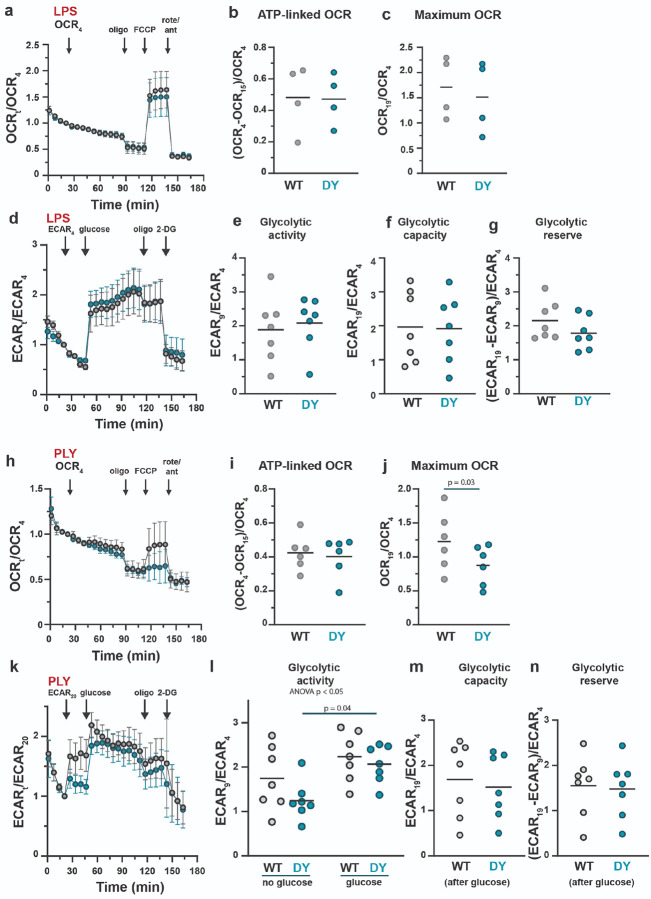
Mitochondrial metabolism is similar in WT and DY BMDMs after LPS stimulation, while OXPHOS is reduced and glycolysis is glucose-dependent in DY BMDMs after PLY stimulation. **(a)** Normalized OCR of BMDMs, **(b)** Normalized ATP-linked OCR, and **(c)** normalized maximum OCR of BMDMs derived from WT (grey) or DY (blue) mice measured using the Seahorse XF Cell Mito Stress Profile. BMDMs were incubated in XF DMEM media with glucose (10 mM), pyruvate (1 mM), and glutamine (2 mM). LPS was added after the fourth cycle (OCR_4_). were derived from profiles shown in (a). **(d)** Normalized ECAR profile, **(e)** glycolytic activity, **(f)** glycolytic capacity, and **(g)** glycolytic reserve BMDMs derived from WT (grey circles) or DY (blue circles) mice, measured using the Seahorse XF Cell Glycolysis Stress Test. BMDMs were initially incubated in XF DMEM with glutamine (2 mM). LPS was added after the fourth cycle (ECAR_4_). Glucose was added after the 8^th^ cycle (ECAR_8_). **(h)** Normalized OCR of BMDMs derived from WT (grey) or DY (blue) mice measured using the Seahorse XF Cell Mito Stress Profile, as in (a). PLY was added to cells after the fourth cycle (OCR_4_). **(i)** Normalized ATP-linked OCR, and **(j)** normalized maximum OCR were derived from profiles shown in (a). **(k)** Normalized ECAR profile, **(l)** glycolytic activity, **(m)** glycolytic capacity, and **(n)** glycolytic reserve of PLY-stimulated BMDMs derived from WT (grey) or DY (blue) mice, measured using the Seahorse XF Cell Glycolysis Stress Test, as in (d). PLY was added to cells after the fourth cycle (ECAR_4_). Glucose was added after 8^th^ cycle (ECAR_8_). **(a, d, h, k)** OCR or ECAR at each time point (OCR_t_ or ECAR_t_) is normalized to OCR or ECAR obtained during the 4^th^ cycle (OCR_4_ or ECAR_4_). Arrows indicate time when inhibitors were added. Symbols represent mean ± SEM of 4, 6 or 7 independent experiments. **(b, c, e, f, g, I, j, l, m, n)** Each symbol represents the average of technical duplicates obtained in 4, 6 or 7 independent assays. **(b, c, e, f, g, i, j, m, n)** Data show values with line at mean, analyzed by paired Student’s t-test. **(l)** Four groups were analyzed by ANOVA followed by pairwise comparison of pre-selected groups: WT vs DY no glucose; WT vs DY with glucose; WT no glucose vs WT with glucose; DY no glucose vs DY with glucose. Significant p-values between paired comparisons are shown.

Next, we quantified how expression of COQ6.DY regulated macrophage mitochondrial metabolism during inflammation. We first quantified the OCR (Fig. 3a, b, c) and the glycolytic activity and capacity (Fig. 3d, e, f) of LPS-stimulated BMDMs from WT or DY mice. For both assays, BMDMs were exposed to LPS 60 min (10 cycles) after initiation of the assay (Fig. 3a, d). During the Glycolysis Stress, BMDMs were incubated in medium with glutamine only for the first 48 min (8 cycles) after assay initiation with the last 24 min (4 cycles) inclusive LPS stimulation). After LPS stimulation, glucose was added as a substrate (Fig. 3d). No differences in the OCR and ECAR profiles, ATP-linked OCR, maximum OCR, glycolytic activity or glycolytic capacity were observed between BMDMs from WT or DY mice following LPS stimulation (Fig. 3a–g).

Although LPS is commonly used to model inflammation, it does not accurately represent inflammatory responses triggered by Gram-positive organisms. We therefore measured OCR, glycolytic activity and glycolytic capacity of BMDMs derived from WT or DY mice following exposure to PLY. Metabolic remodeling after PLY stimulation was distinct from that induced by LPS (Fig. 3h–n). There was no difference in ATP-linked OCR between BMDMs from WT or DY mice (Fig. 3i), but the maximum OCR of DY BMDMs was significantly reduced compared to WT (Fig. 3j). Furthermore, WT BMDMs upregulated glycolysis immediately after exposure to PLY in the absence of glucose, with a second increase after glucose was added (Fig. 3k). In contrast, PLY-stimulated DY BMDMs did not upregulate glycolysis until after glucose was added (Fig. 3l). We noted no difference in glycolytic capacity or reserve when glucose became available (Fig. 3m, n).

Comparison of metabolic energy profiles of WT BMDMs stimulated by LPS or PLY compared to the baseline profile exhibited a change in the “slope” of the regression lines, showing a downregulation of OXPHOS and shift towards glycolysis. In contrast, the “slope” of DY BMDMs stimulated by LPS or PLY shows a relatively conserved reliance on glycolysis regardless of inflammatory state (**Suppl. Figure 1**).

**Figure 4:**
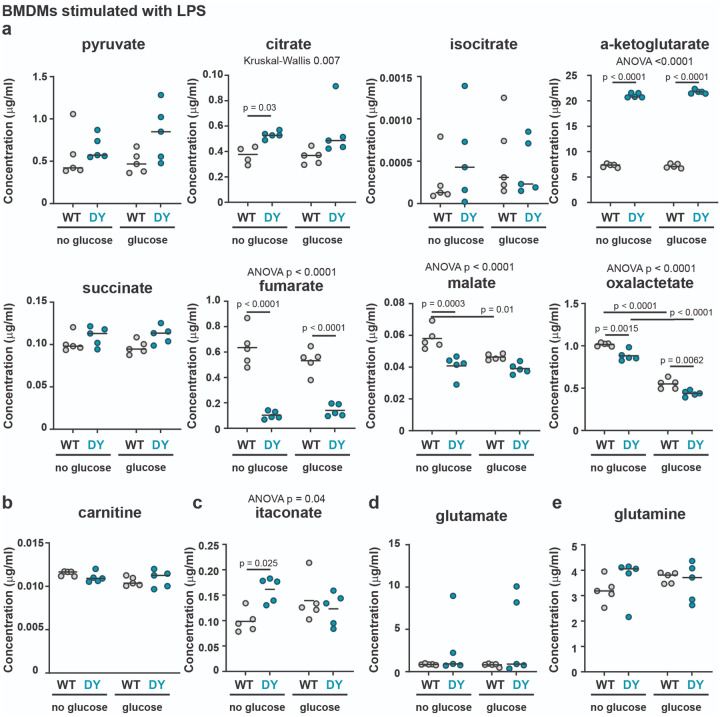
Key TCA metabolites altered in BMDMs from DY mice following LPS stimulation. **(a)**Concentrations of pyruvate and TCA cycle intermediates, (b) carnitine, (c) itaconate, (d) glutamate, or (e) glutamine in LPS-stimulated BMDMs derived from WT (gray) or DY (blue) mice, incubated with or without glucose. Each symbol represents value of one of five replicates of BMDMs pooled from at least three mice. Line at mean for normally distributed data or median for non-parametric data. Four groups in each graph were analyzed by either ANOVA (normally distributed) or by Kruskal-Wallis (non-parametric data), followed by pairwise comparison of pre-selected groups: WT vs DY no glucose; WT vs DY with glucose; WT no glucose vs WT with glucose; DY no glucose vs DY with glucose. Significant p-values between paired comparisons are shown.

**Figure 5:**
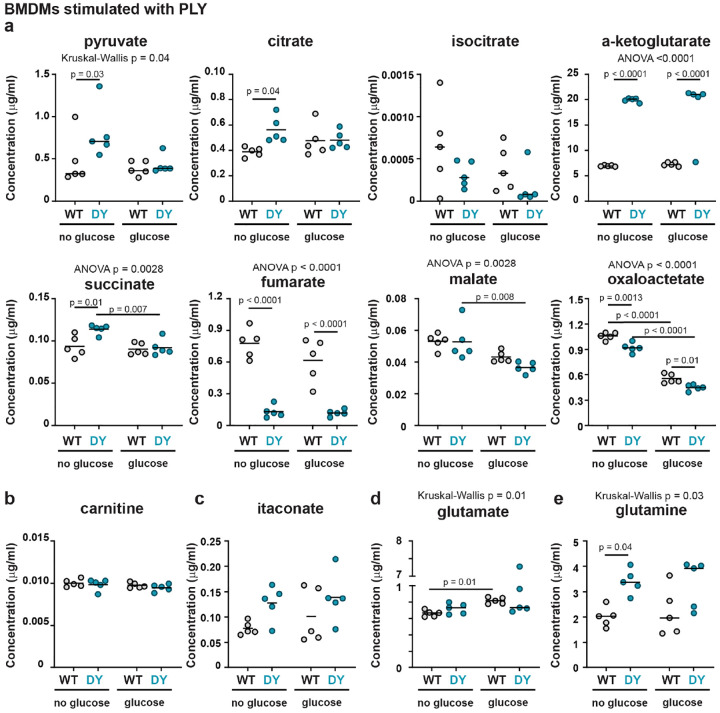
Key TCA metabolites altered in BMDMs from DY mice following PLY stimulation. **(a)** Concentrations of pyruvate and TCA cycle intermediates, **(b)** carnitine, **(c)** itaconate, **(d)**glutamate, or **(e)** glutamine in PLY-stimulated BMDMs derived from WT (gray) or DY (blue) mice, incubated with or without glucose for 1 h. Each symbol represents value of one of five replicates of BMDMs pooled from at least three mice. Line at mean for normally distributed data or median for non-parametric data. Four groups in each graph were analyzed by either ANOVA (normally distributed) or by Kruskal-Wallis (non-parametric data), followed by pairwise comparison of pre-selected groups: WT vs DY no glucose; WT vs DY with glucose; WT no glucose vs WT with glucose; DY no glucose vs DY with glucose. Significant p-values between paired comparisons are shown.

We next quantified pyruvate, TCA substrates, carnitine, itaconate, glutamine and glutamate concentrations in BMDMs derived from WT and DY mice after stimulation with either LPS (Fig. 4) or PLY (Fig. 5), with or without glucose. Significant increases in α-ketoglutarate and decreases in fumarate concentrations were again observed in BMDMs from DY mice (Fig. 4a, 5a), regardless of glucose availability. After LPS exposure, citrate and itaconate were elevated in BMDMs from DY mice, compared to WT, in the absence of glucose (Fig. 4a, c). Malate was elevated in WT compared to DY in the absence of glucose. Incubation in glucose resulted in downregulation of malate in WT cells but had no effect on malate concentrations in DY cells. Finally, oxaloacetate concentrations varied by genotype and glucose availability, with BMDMs from DY mice consistently exhibiting reduced oxaloacetate compared to WT, and with both WT and DY BMDMs revealing downregulated oxaloacetate in the presence of glucose. (Fig. 4a). Thus, LPS stimulation significantly altered concentrations of TCA metabolites in BMDMs derived from DY mice, even while no differences in OXPHOS or glycolysis remodeling were observed (Fig. 4).

After PLY stimulation, BMDMs from WT and DY mice expressed significantly different concentrations of the following metabolites: pyruvate, glutamine, and all TCA metabolites except isocitrate (Fig. 5). Specifically, increased α-ketoglutarate and reduced fumarate were observed independent of glucose availability (Fig. 5a). Pyruvate, citrate, succinate and glutamine concentrations were elevated in DY BMDMs compared to WT when glucose was absent but were equivalent when glucose was present (Fig. 5a, e). Glucose availability reduced the concentrations of succinate and malate and increased that of glutamate in BMDMs from DY mice, but not those from WT. Finally, BMDMs from DY mice exhibited lower concentrations of oxaloacetate compared to WT independently of glucose availability, while glucose availability downregulated oxaloacetate in both WT and DY BMDMs (Fig. 5a). These many differences in TCA metabolite concentrations after PLY exposure reveal that inflammatory remodeling of macrophage metabolism depends upon inflammatory stimulus, *Coq6* genotype, and glucose availability.

**Figure 6:**
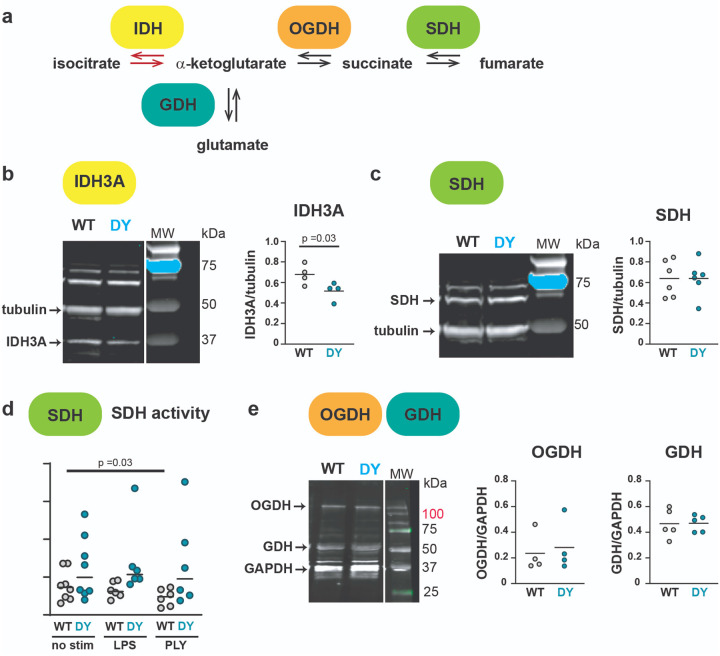
Expression of IDH3A reduced in BMDMS derived from DY mice. **(a)** Schematic showing assayed enzymes that participate in the TCA cycle. **(b)** Representative immunoblot and quantification of protein expression of IDH3A and **(c)** SDH. Protein densities were normalized to tubulin. **(d)** SDH activity exhibited by BMDMs from WT (gray) or DY (blue) mice with or without stimulation by LPS or PLY. Each symbol represents value from one of 6 or 8 independent experiments, line at mean, p-value determined by paired t-test. **(e)** Representative immunoblot and quantification of protein expression of OGDH and DH, normalized to GAPDH. **(b, c, e)** The full immunoblots from which images were cropped are provided in **Suppl. Fig. 2.** The molecular weight (MW) standard for (b) and (c) is cropped from the same gel, as shown in Suppl. Fig. 2, and as indicated by the space between the IDH3A immunoblot and the MW markers. Each symbol represents relative protein expression of indicated enzyme from BMDMs derived from WT (gray) or DY (blue) obtained from one of 4 or 5 independent experiments. Line shown at mean (normally distributed) or median (non-parametric distribution) with p-values determined by Student’s t-test (normally distribution) or Mann-Whitney (non-parametric distribution).

We next evaluated if altered expression of enzymes in the TCA cycle could explain the elevation of α-ketoglutarate and/or reduced fumarate in DY BMDMs. The conversion of isocitrate to α-ketoglutarate is catalyzed by isocitrate dehydrogenase (IDH) and represents the rate-limiting step of the TCA cycle (Fig. 6a). Oxoglutarate dehydrogenase (OGDH) converts α-ketoglutarate to succinate, followed by conversion of succinate to fumarate by succinate dehydrogenase (SDH). Glutamine, which can serve as an alternate energy source, feeds into the TCA cycle after conversion to glutamate, followed by glutamate conversion into α-ketoglutarate by glutamate dehydrogenase (GDH). To determine if increased α-ketoglutarate in DY BMDMs was driven by increased expression of IDH, we quantified expression of IDH3A by immunoblot (Fig. 3b). Expression of IDH3A was modestly, but significantly, downregulated in BMDMs derived from DY mice. Downregulation of IDH3A is consistent with negative feedback from excess α-ketoglutarate, but does not explain why α-ketoglutarate is present at high levels. We then quantified SDH protein expression by immunoblot to determine if reduced fumarate levels in DY BMDMs could be due to decreased SDH expression or activity; however, SDH expression was equivalent between WT and DY BMDMs (Fig. 6c). Activity of SDH in WT BMDMs declined after PLY stimulation, while SDH activity was equivalent in DY BMDMs before and after PLY stimulation (Fig. 6d). No difference in SDH activity between WT and DY BMDMs was observed. Finally, we assessed if altered expression of either OGDH or GDH was associated with increased α-ketoglutarate. OGHD and GDH were expressed at similar levels in WT and DY BMDMs (Fig. 6e). Full immunoblots are provided in **Suppl. Figure 2.**Thus, the different concentrations of α-ketoglutarate, fumarate and glutamine in WT and DY BMDMs could not be explained by altered expression of TCA enzyme expression. Expression of COQ6.DY must therefore alter relative abundance of cellular metabolites through mechanisms other than enzyme expression levels.

**Figure 7:**
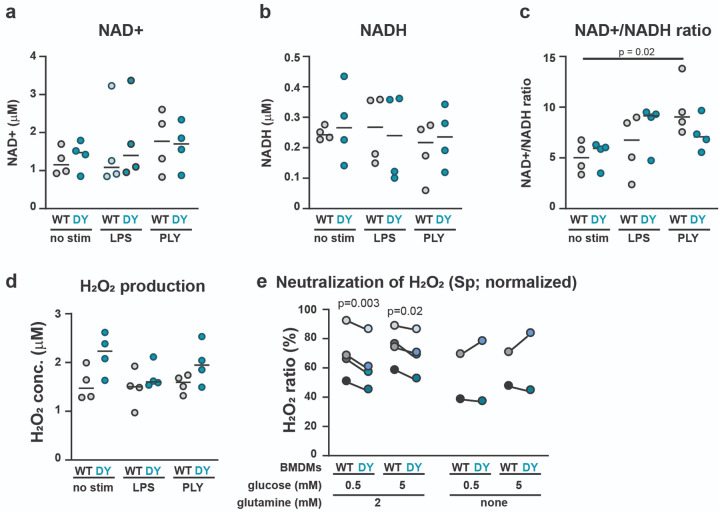
Regeneration of NADH and neutralization of *S. pneumoniae*-generated H_2_O_2_ in WT or DY BMDMs. **(a)** Total NAD+, **(b)**, total NADH, and **(c)** NAD+/NADH ratio quantified from BMDMs from WT (gray) or DY (blue) after incubation with vehicle control, LPS or PLY for 60 min. Each symbol represents a replicate value from 1 of 2 independent experiments (biological replicates). Line at median, p-value determined by Mann-Whitney. **(d)** H_2_O_2_ production generated by BMDMs from WT (gray) or DY (blue) mice after incubation with LPS, PLY or vehicle control for 1 h. Each symbol shows average of technical duplicates from 1 of 4 independent experiments, line at median. **(e)** Neutralization of Sp-generated H_2_O_2_ by BMDMs from WT (gray) or DY (blue) mice. To compare multiple biological experiments, we show data normalized to the total H_2_O_2_ generated by *S. pneumoniae* in each experiment. Neutralization (%) calculated as 100 * (1 - H_2_O_2_ measured in infected cells/ H_2_O_2_ measured in *S. pneumoniae* only sample). Each symbol shows average of technical duplicates from 1 of 4 independent experiments. P-values determined by paired t-tests.

We have previously documented that BMDMs from DY mice exhibit impaired ROS production and reduced intracellular *S. pneumoniae* killing ^[4]^. To identify pathways that could be altered by aberrant metabolic remodeling and result in impaired bacterial killing, we measured cytokine production (**Suppl. Figure 3**) and NAD + and NADH concentrations (Fig. 7a–c), in LPS- or PLY-stimulated BMDMs from WT or DY mice. We also measured the neutralization of pneumococcal-derived hydrogen peroxide (Fig. 7d,e). Cytokine production was measured from WT or DY BMDMs incubated in media with or without glucose 3 h after LPS or 1h after PLY exposure. We selected a short time course to coincide with the rapid changes in metabolism observed (e.g. Figure 3a,d,h,k). Significant differences in cytokine production varied by stimulus, but not by *Coq6* genotype or glucose availability. Specifically, LPS induced greater production of IL-6, IL-12, IL-10, CCL3, CCL4, CCL5, CXCL1 and CXCL10 than did PLY. The early differences in metabolism between WT and DY BMDMs did not change early cytokine release.

The TCA cycle regenerates NADH from NAD+, ensuring availability of reduced NADH iof isocitrate to α-ketoglutarate, conversion of α-ketoglutarate to succinyl-CoA, and conversion of malate to oxaloacetate. To determine if different relative abundances of TCA metabolites in BMDMs from DY mice correlated with differences in the ratio of NAD + to NADH, we quantified the concentrations of NAD + and NADH, and the NAD+/NADH ratio, in BMDMs from WT and DY mice that were unstimulated, LPS-stimulated, or PLY-stimulated (Fig. 7a–c). Under homeostatic conditions and after LPS exposure, there were no differences in the NAD+/NADH ratio between WT and DY BMDMs. However, PLY stimulation significantly increased the NAD+/NADH ratio in BMDMs from WT mice, while no significant elevation in BMDMs from DY mice was observed (Fig. 7c). BMDMs from WT mice thus created an increased oxidizing environment in response to PLY, while BMDMs from DY mice did not.

Finally, we assessed hydrogen peroxide (H_2_O_2_) from BMDMs after stimulation and the neutralization of pneumococcal-generated H_2_O_2_ (Fig. 7d, e). The generation of mitochondrial reactive oxygen species (mtROS) is one mechanism by which macrophages eliminate pathogens, and mtROS production is generated via OXPHOS. H_2_O_2_ is a major species of mtROS. However, we found that neither WT nor DY BMDMs generated measurable quantities of H_2_O_2_ after stimulation with either LPS or PLY (Fig. 7d), indicating that the rapid changes in OXPHOS metabolism did not immediately correlate with changes in H_2_O_2_ production.

Sp produces significant levels of H_2_O_2_ to promote colonization and to serve as a major virulence factor during infection ^[7]^. Macrophages neutralize Sp-generated H_2_O_2_ through the action of enzymes such as catalase and glutathione peroxidase ^[8, 9]^. We compared the ability of WT and DY BMDMs to neutralize H_2_O_2_ 1 h after exposure to Sp when incubated at low (0.5 mM) or high (5 mM) concentrations of glucose and with (2 mM) or without glutamine. In the presence of glutamine, WT BMDMs neutralized significantly more H_2_O_2_ than did DY cells, irrespective of glucose concentrations (Fig. 7e). In the absence of glutamine, we observed no differences between H_2_O_2_ neutralization.

## Discussion

We previously showed that a variant in *Coq6* accelerated the downregulation of OXPHOS in response to pneumococcal infection ^[4]^. We undertook this study to systematically analyze how *Coq6*^*DY*^ expression reprograms macrophage metabolic remodeling in response to two different pro-inflammatory challenges (LPS or PLY), in the context of variation in substrate availability.

Macrophages rapidly undergo significant metabolic remodeling when shifting from a resting to an activated state ^[3]^. Macrophage activation states exist across a spectrum, with the two extremes historically represented as M1 (pro-inflammatory) and M2 (pro-healing). The M1 state is frequently induced *in vitro* by stimulating macrophages with LPS and IFN-γ, while M2 is induced by exposure to IL-4 and IL-13. Remodeling of mitochondrial metabolism supports and accompanies macrophage polarization. M1 macrophages exhibit down-regulated OXPHOS and upregulated aerobic glycolysis, while M2 macrophages exhibit greater reliance upon OXPHOS, increased fatty acid oxidation and glutaminolysis ^[1]^. The shift from OXPHOS to glycolysis is accompanied by diversion of TCA cycle metabolites, leading to the accumulation of succinate, citrate, and itaconate. Increases in succinate, fumarate and other metabolites act to sustain an inflammatory phenotype ^[10]^. For example, increased succinate availability stabilizes the transcription factor HIF-1α, which upregulates a genetic program necessary to support an inflammatory phenotype, including anti-apoptotic genes, pathways supporting glycolysis, and immunoregulatory receptors such as PD-1 and PD-L1 ^[3, 11]^.

Categorization of macrophages into distinct M1 or M2 states induced by cocktails of polarizing cytokines insufficiently captures the spectrum of macrophage phenotypes found during inflammation *in vivo*. To better replicate macrophage phenotypes relevant to *in vivo* infection with either a Gram-positive bacterium (*S. pneumoniae*) or a Gram-negative bacterium, we compared BMDMs activated by PLY or LPS. LPS is a pathogen-associated molecular pattern (PAMP) universal to infections with Gram-negative bacteria that binds to Toll-like receptors and induces NF-κB signaling. PLY is a pore-forming toxin derived from the Gram-positive organism *S. pneumoniae* that, at sub-lytic concentrations, triggers NLRP3 inflammasome activation and may suppress macrophage metabolic remodeling ^[12]^. In our study, both LPS and PLY activated WT BMDMs, as assessed by induction of inflammatory cytokines and chemokines (**Suppl. Figure 3**), but elicited distinct phenotypes. LPS induced significantly higher concentrations of some inflammatory mediators, such as IL-6, CCL4 and CXCL10, than did PLY (**Suppl Fig. 3a, b**). LPS and PLY increased the glycolytic activity of WT BMDMs. PLY, but not LPS, significantly downregulated OXPHOS in the short timeframe of our analyses. LPS stimulation increased, while PLY reduced, the relative concentrations of glutamine in WT BMDMs. PLY increased the NAD+/NADH ratio in WT BMDMs (Fig. 7c), while LPS did not. Our results highlight the plasticity of macrophage responses to different PAMPs, and reinforce the need to evaluate macrophage activation to multiple pro-inflammatory stimuli.

Many analyses of macrophage mitochondrial metabolism use standardized concentrations of glucose (5 or 10 mM), pyruvate (1 mM) and glutamine (2 mM) ^[13]^. However, glucose concentrations in fluid lining airway epithelial surfaces may be as low as 0.4 mM in healthy lungs, while pathologic conditions such as cystic fibrosis, diabetes, or chronic inflammation can increase airway glucose to 2–4 mM ^[14]^. Elevated glucose concentrations *in vivo* can spur growth of pulmonary pathogens such as *Staphylococcus aureus*, *Pseudomonas aeruginosa*, and *S. pneumoniae*, increasing the likelihood of infection ^[5, 14]^. *S. pneumoniae* relies heavily on glycolysis for energy production and prefers glucose as a source. After onset of pneumococcal infection, airway glucose concentrations can drop precipitously as dividing bacteria consume available glucose ^[5]^. We therefore also compared the effects of no or low (0.5 mM) glucose on macrophage metabolic remodeling to those found at “high” (5 mM) glucose. Glycolytic activity in WT BMDMs after PLY stimulation did not vary with presence of glucose. In LPS-stimulated WT BMDMs, TCA substrates malate and oxaloacetate decreased with increased glucose availability, while PLY-stimulated WT BMDMs exhibited reduced oxaloacetate and increased glutamate.

**Figure 8:**
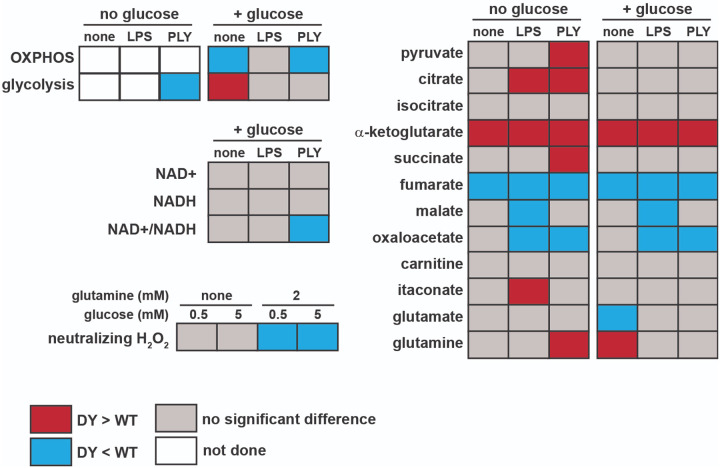
Summary of observed metabolic differences between BMDMs from WT or DY mice under different conditions. Red indicates that the measured parameter is greater in DY than WT BMDMs, blue indicates that DY is reduced compared to WT, gray indicates equivalence, and an open box indicates that the parameter was not measured.

The DY genotype significantly altered mitochondrial metabolic remodeling, relative concentrations of TCA cycle intermediates, the NAD+/NADH ratio and neutralization of pneumococcal-generated H_2_O_2_ at homeostasis and after stimulation with either LPS or PLY (summarized in Fig. 8). At homeostasis, with glucose, BMDMs from DY mice exhibited greater reliance on glycolysis and reduced OXPHOS, with increased accumulation of α-ketoglutarate and glutamine and reduced fumarate and glutamate (Fig. 8). No differences in OXPHOS or glycolysis were observed in BMDMs from DY (compared to WT) after LPS stimulation (with glucose). Concentrations of α-ketoglutarate remained higher in DY compared to WT after LPS stimulation, with increases in citrate and itaconate also seen in the absence of glucose. Concentrations of fumarate, malate, and oxaloacetate were lower in DY BMDMs following LPS stimulation, regardless of glucose availability.

Compared with LPS, PLY stimulation revealed more significant differences between metabolic remodeling in BMDMs from DY (compared to WT) mice, and, in some cases, a greater dependence of DY BMDMs on glucose. For instance, WT BMDMs upregulated glycolysis in response to PLY regardless of glucose concentration, while DY BMDMs upregulated glycolysis in response to PLY only when glucose was present. OXPHOS was significantly downregulated in DY BMDMs after PLY stimulation, as we previously observed ^[4]^. In the presence of glucose, α-ketoglutarate was increased and fumarate and oxaloacetate were reduced in PLY-stimulated DY BMDMs compared to WT. In the absence of glucose, α-ketoglutarate, pyruvate, citrate, and succinate were increased in PLY-stimulated DY BMDMs, while fumarate and oxaloacetate remained reduced.

To study the impacts of *Coq6*^*DY*^ on pathways required for bacterial killing, we measured NAD+/NADH ratios in BMDMs from WT or DY mice after stimulation with LPS or PLY, and we measured the ability of BMDMs to neutralize or suppress *S. pneumoniae*-generated H_2_O_2_. The TCA cycle regenerates NADH for use as an electron donor in the electron transport chain. Increased NAD + in host cells, and a corresponding increase in the NAD+/NADH ratio indicating a more oxidizing environment, has been correlated with reduced pneumococcal growth and improved anti-bacterial host defense ^[15]^. We saw no significant differences in NAD+, NADH, or the NAD+/NADH ratio in BMDMs from WT or DY mice after LPS stimulation (with glucose). After PLY stimulation, BMDMs from WT mice exhibited a more oxidized environment (increased NAD+/NADH ratio) compared to baseline. In contrast, there was no significant difference in the NAD+/NADH ratio in BMDMs from DY mice at homeostasis or after PLY stimulation. The ability of WT BMDMS, but not DY BMDMs, to increase the NAD+/NADH ratio in response to PLY correlates with our prior observation that BMDMs from WT mice have greater ability to kill intracellular *S. pneumoniae* ^[4]^.

Another critical factor produced during the *S. pneumoniae*:macrophage interaction is H_2_O_2_. The role of H_2_O_2_ during pneumococcal infection is complex, as *S. pneumoniae*-produced H_2_O_2_ contributes to pneumococcal virulence, while H_2_O_2_ produced by macrophages can promote pneumococcal clearance. An important host defense mechanism against *S. pneumoniae*-generated H_2_O_2_ is the production of antioxidants including glutathione peroxidase, for which glutamine serves as an essential precursor ^[16, 17]^. Glutamine also serves as a major carbon and nitrogen source and supports a pro-inflammatory state in macrophages ^[18]^. Conversion of glutamine to glutamate and subsequent conversion of glutamate to α-ketoglutarate supports the TCA cycle and contributes to the generation of itaconate ^[19]^. Macrophages increase reliance upon glutamine when glucose is unavailable ^[20]^. Because airway glucose concentration is low at later timepoints in experimental *S. pneumoniae* infection, and macrophages may then rely more upon glutamine ^[5, 20]^, we tested the ability of BMDMs to neutralize or suppress Spn-generated H_2_O_2_ in the presence or absence of glutamine (Fig. 7e). Neutralization of H_2_O_2_ was reduced in DY BMDMs when assayed in the presence of glutamine. In the absence of glutamine, the ability of BMDMs from WT or DY mice to suppress Sp-generated H_2_O_2_ was equivalent, regardless of glucose concentration. Variation in the dependence of metabolic remodeling on macrophage genotype and substrate availability aligns with a prior report that glutamine metabolism varies with macrophage lineage ^[21]^.

The observation that BMDMs from DY mice consistently exhibit increased α-ketoglutarate and reduced fumarate concentrations, regardless of stimulation condition or glucose availability, generates several hypotheses for future study. Increased α-ketoglutarate and reduced fumarate cannot be explained by differential expression of the TCA cycle enzymes isocitrate dehydrogenase, oxoglutarate dehydrogenase, glutamate dehydrogenase, or succinate dehydrogenase, nor by reduced activity of succinate dehydrogenase. The equivalent concentrations of malate in WT and DY BMDMs, despite significantly reduced fumarate, suggests that malate concentration is being maintained from another substrate, such as pyruvate. Reduced levels of fumarate, without changes in concentrations of the precursor succinate, suggests that fumarate is being redirected into another metabolic pathway. Increased concentrations of α-ketoglutarate and variations in the relative levels of glutamate and glutamine suggest possibilities such as differential uptake of glutamine or differential regulation of glutamate metabolism, such as changes in the urea cycle, generation of glutathione, or purine biosynthesis.

The intent of this study was to provide sufficient characterization of metabolic remodeling in a model cell derived from WT or DY mice, to determine relevant outcomes, stimuli and metabolites for future in-depth analyses. Limitations of this study therefore include a reliance upon *in vitro* studies of BMDMs, a model cell selected because large numbers are easily obtainable for the assays required. BMDMs may not reflect the metabolic changes in other macrophage lineages such as alveolar macrophages. Additionally, BMDMs may not reflect other tissues of interest, such as cardiac tissue. Also, we measured relative concentrations of TCA and other essential metabolites but have not yet tested metabolic flux through use of labeled compounds and following carbon incorporation over time.

In summary, we conclude that macrophage metabolic remodeling varies with substrate availability, with host genotype, and with the type of inflammatory stimulus received. Our study is consistent with a prior report that metabolic remodeling can vary with macrophage lineage ^[21]^. Differential remodeling by BMDMs from WT or DY mice in response to PLY further suggests that the DY genotype may restrict the flexibility of macrophages to use different carbon sources during inflammation. Specifically, BMDMs from DY mice did not upregulate glycolysis in response to PLY until glucose was provided, whereas BMDMs from WT mice upregulated glycolysis with or without glucose. Our observation that metabolic remodeling depends upon glucose availability is critical, because glucose availability during *in vivo* bacterial infections varies widely by pathogen, tissue, and time after infection. The pleiomorphic inflammatory responses of macrophages may therefore be driven *in vivo* by the rapidly changing inflammatory milieu.

## Conclusions

Our newly discovered SNV in *COQ6*, *COQ6*^*DY*^, fundamentally alters macrophage mitochondrial metabolism, illuminating how macrophage metabolic status and energy source usage varies during inflammation.

## Supplementary Material

Supplementary Files

This is a list of supplementary files associated with this preprint. Click to download.

- SupplementaryFiguresforsubmissionRevisedbyXL.pdf

## Data Availability

No large datasets were generated for this publication. All data are available upon request.

## Key points

Macrophage metabolic remodeling varies with genotype, glucose availability and inflammatory stimulus.

## Data Availability

No large datasets were generated for this publication. All data are available upon request.

## Abbreviations

BMDMs: bone marrow-derived macrophages
DY: homozygous for *Coq6*^*DY*^ expression
ECAR: extracellular acidification rate
GDH: glutamate dehydrogenase
IDH3a: isocitrate dehydrogenase
LPS: lipopolysaccharide
OCR: oxygen consumption rate
OGDH: oxoglutarate dehydrogenase
OXPHOS: oxidative phosphorylation
PLY: pneumolysin
SDHA: succinate dehydrogenase
SNV: single nucleotide variant
TCA: tricarboxylic acid
WT: wild-type
